# How do people experience innocent suffering?

**DOI:** 10.3389/fpsyg.2023.1148902

**Published:** 2023-06-26

**Authors:** Evgeny Smirnov

**Affiliations:** 4xxi Software Ltd., Richmond, Surrey, United Kingdom

**Keywords:** belief in a just world, innocent suffering, justice, causal coherence, critical event

## Abstract

**Introduction:**

The paper examines the psychological facet of innocent suffering. One can find a description of this phenomenon in social psychology as a factor that affects the belief in a just world, but there is a lack of qualitative scientific data about related psychological features, processes, copings, and consequences on the personality level.

**Methods:**

To study innocent suffering, semi-structured in-depth interview was conducted (31 respondents, ~223 minutes per respondent, 6,924 min in total) aimed to gather data about the experiences of innocent sufferings happened to participants. For the analysis of texts, a narrative and content analysis are used within the framework of grounded theory. The reliability of the results is based on expert assessment.

**Results and discussion:**

As a result, six essential properties of innocent sufferings were identified: complexity, stability, distress, injustice, casual incoherence, and breaks of integrity of a life story. The most “popular” life domains, in which participants reported about innocent sufferings, are violence, abuse (physical and psychological), and quitting romantic relationships. It is proposed a scientific definition of innocent suffering and the prototype of the phenomenon.

## 1. Introduction

The phenomenon of innocent suffering has been well-known for centuries. From the literature of Ancient Mesopotamia, one might find narratives of a suffering hero, who does not understand the reasons for the events that are happening to him (Mattingly, 1990; Foster, 2005; Oshima, 2014). Mattingly (1990, p. 318-9) and Clifford (2007, p. 8) emphasize five most known poems about this theme written between 2000 and 1000 BC: Sumerian “Man and His God” (Mattingly, 1990), “The Dialogue between a Man and His God” (Mattingly, 1990), “The Sufferer's Salvation” (Bricker, 2000), “Ludlul bēl nēmeqi” (“Let me praise the Lord of Wisdom”) (Pritchard, 1969; Mattingly, 1990; Foster, 1996), and “The Babylonian Theodicy” (Clifford, 2007).

In every poem, the hero tries to realize why he suffers. Eventually, he finds a plausible explanation, which ends suffering and restores health and wellbeing.^1^ The structure of such narratives is almost always the same: (1) a description of sufferings, (2) complaints about sufferings, and (3) healing and restoration (Gese, 1958; Hallo and Younger, 2003). The motif of the innocent sufferer reaches its climax in the well-known Book of Job, which describes a story of a pious man who, as a result of a dispute between Yahweh and Satan, loses his wealth, health, and family without committing any sin (Smirnov, 2021).

The core psychological conflict causing distress that faces the heroes of these poems is grounded in their religious and traditional beliefs. The protagonists have a justified (by their tradition or religion) belief that if one is pious and has no sins, one deserves happiness and wellbeing. When this is not the case, sufferings arise. Philosophers call this conflict *the problem of evil*. Hume (1779) introduces the following reasoning for this problem:

Is [God] willing to prevent evil, but not able? Then is he impotent? Is he able, but not willing? Then is he malevolent?

While it is up to an individual whether or not to believe in God, there is a psychological fact of the existence of these sufferings. Moreover, conflict arises even for those people who are not theists. In psychology, one might connect innocent suffering to the broader *belief in a just world* (Lerner and Matthews, 1967; Rubin and Peplau, 1973, 1975; Lerner and Miller, 1978; Lerner, 1991; Dalbert, 1999; Hafer and Begue, 2005; Ellard et al., 2016; Hafer and Sutton, 2016; Dawtry et al., 2020). It states that “*[i]ndividuals have a need to believe that they live in a world where people generally get what they deserve*” (Lerner and Miller, 1978, p. 1030).

Some authors claim that innocent sufferings are one of the possible threats to this belief (i.e., Lerner and Matthews, 1967; Lerner and Miller, 1978; Maes and Schmitt, 1999; Hafer and Begue, 2005; Correia et al., 2007; Hafer and Gosse, 2011; Hafer and Sutton, 2016). When one participates in or observes such an event without a plausible justification of what is going on, one experiences a dissonance that leads either to suffering or to the activation of some defense mechanisms, for example, derogation of a victim (in case of observing) or finding acausal reasons, such as sins or a [divine] test (Hafer and Gosse, 2011; Hafer and Sutton, 2016; Smirnov, 2021).

Not every event that looks unjust is considered by observers as unjust. Mikula (1993, pp. 229-30) emphasizes five characteristic features of injustice: it should (1) violate an entitlement, (2) be caused by another agent, who (3) has some control over the situation (can inflict damage but can avoid it), (4) has the intention to perform the unjust action, and (5) has no plausible justification to perform it.

The belief in a just world is not only a cognitive construct. It seems to be a psychological need required for wellbeing (Lipkus et al., 1996; Hafer and Sutton, 2016). Lerner and Miller (1978, p. 1030) claim that one cannot focus on long-term goals or have socially regulated behavior without this belief. Hence, any serious threat to it requires one's response.

One might notice that many experimental studies investigating the belief in a just world have a design that implies respondents watch, read, or listen to unjust events happening to others. For example, Dawtry et al. (2020) performs the meta-analysis of 55 cases, none of which is related to one's own experience of innocent suffering. Moreover, researchers (i.e., Rubin and Peplau, 1973; Lerner, 1991; Hafer and Sutton, 2016) often focus only on the consequences of one's interaction with the experience of innocent suffering, such as derogation of victims or other cognitive distortions about the world or people.

While such experiments show interesting results, they do not provide any insights into the experience of innocent suffering itself because the priming stimulus is weak. Moreover, Lerner and Miller (1978) notice that overall, people do not believe that the world is just. The function of the belief in a just world is to explain what is happening to oneself or close people. Accepting injustice against others does not seriously threaten the belief (Lerner and Miller, 1978).

Therefore, if one wants to study innocent suffering, one should collect data about personal experiences. The aim of this paper is to describe the content of the experience of innocent suffering. In other words, how do people experience innocent suffering, what are the categories of life events that might trigger such experiences, how do they cope with them, and what are the core features that distinguish innocent sufferings from other sufferings or threats to the belief in a just world?

## 2. Materials and methods

### 2.1. Methodology

To answer the questions of what innocent sufferings are and how people experience and cope with them, it is reasonable to use qualitative methods (Grishina, 2018). While there are other psychological constructs that could be related to this phenomenon, such as the belief in a just world (Lerner and Miller, 1978), justice and injustice (Mikula, 1993), and existential problems (Yalom, 1980), for this study, the method of the grounded theory developed by Glaser and Strauss (1967) was employed. This method, initially used in sociology, has since become widely used in psychology (Henwood and Pidgeon, 2003). As a methodology, grounded theory aims to conceptualize, structure, and describe a phenomenon and construct a theory based on a series of methods. The core difference from the traditional approach is that hypotheses are defined during the study rather than in the beginning (Charmaz, 1996, 2014). The researcher collects and analyzes data at the same time and makes, accepts, or rejects assumptions all the time. For example, in this study, unlike the classical approach, the definition of the term “innocent suffering” is not set a priori. On the contrary, the aim of this study is to build such a definition. The sufficiency of the sample is assessed not on the basis of generally accepted quantitative indicators but based on so-called theoretical sampling, which is the process of meaningful selection of the cases needed to be analyzed (Charmaz, 1996, 2014). Grounded theory analysis involves coding text and theorizing, writing running notes (memoing) to identify concepts, and integrating, refining, and writing up theories. This is done through the constant comparative method and negative case analysis. Memoing contributes to theory building by providing an intermediate step between coding and writing up the analysis (Glaser and Strauss, 1967).

There is a number of studies in psychology that are using the framework of grounded theory. It is especially useful when one wants to describe a phenomenon that involves subjective emotional experiences. For example, Klomek et al. (2015) study bullying and its effects on suicidality and criminality in adulthood. Thornberg et al. (2013) construct a concept of being a victim of bullying under the grounded theory approach. Wilcockson (2020) investigate training, socialization, and adaptation processes when one exits one culture and transitions to another. Yakushko (2010) builds a model of coping strategies used by immigrants. Tuason (2013) describes the psychological experience of poverty in the Philippines. Moreover, it is also possible to study dynamical processes under the framework of grounded theory. For example, Carrero et al. (2000) investigate radical innovation within organizations from the holistic perspective as a complex and continuous process. Overall, these studies show that one might use the framework of grounded theory to describe and analyze integrative, dynamical, highly affective, and controversial phenomena, which is the case for innocent suffering.

### 2.2. Narrative and content analysis of life stories

Many studies of the belief in a just world (and hence, innocent suffering) are focused on the investigation of humans' traits and coping strategies and correlations between them, which corresponds to the first part of the definition of personality suggested by McAdams and Pals (2006, p. 212): “*[p]ersonality is an individual's unique variation on the general evolutionary design for human nature, expressed as a developing pattern of dispositional traits and characteristic adaptations*”. However, there is a second part that is related to the “*integrative life stories complexly and differentially situated in culture*” (McAdams and Pals, 2006, p. 212). To study narratives, one may use qualitative methods, such as narrative and content analysis (McAdams, 2001, 2018; Pals, 2006; Kvale, 2007; Madill and Gough, 2008; Patton, 2015; Lune and Berg, 2017; Grishina, 2018).

Narrative analysis is a method that examines *narratives*—internally coherent life stories. Habermas and Bluck (2000) distinguish four types of coherence in narratives: temporal, cultural, thematic, and causal. The first type represents the temporal order and connections of one's life events. Usually, it is chronological. However, one might sometimes face exceptions. Cultural coherence examines the correspondence of a story to the sociocultural context. Thematic coherence defines an implicit theme of a narrative. For the life path, it unites many events through a shared idea. Habermas and Bluck (2000) emphasize that this idea appears usually near the beginning or end of a story, as well as close to its “turning” points.

Causal coherence means the consistency of the changes in one's identity, circumstances, and events that occurred (Habermas and Bluck, 2000). This type adds meaning to the narrative and makes it consistent, relevant, and self-connected (Trabasso et al., 1995). Any causal incoherence leads to “*life appears to have been determined by chance and therefore to be meaningless*” (Habermas and Bluck, 2000, p. 751). Note that causal coherence explains both action and identity changes. The former requires a consistent and integral sequence of events that plausibly follow each other. The latter implies the plausible explanation of identity changes, i.e., the analysis of past circumstances and events and determining causal links between the old and the current identities (Habermas and Bluck, 2000). It follows that when one studies a narrative, one should pay attention to any incoherence found. Each incoherence requires a plausible explanation. For innocent suffering, causal incoherences are the most important because of their connection to the meaning-making process.

The second method of qualitative analysis of innocent suffering used is a content analysis that allows studying texts, by which I mean here and after any content produced, experienced, or acknowledged by a person, created by respondents and finding their meanings (Hsieh and Shannon, 2005; Erlingsson and Brysiewicz, 2017). In content analysis, texts are split into chunks that are coded, labeled, and grouped into categories and themes. The list of categories can be defined a priori (*directed content analysis*) or a posteriori (*conventional*). One might combine both approaches (*summative content analysis*). In this paper, conventional content analysis is used to identify the features of the experience of innocent suffering because these features are not known a priori.

### 2.3. Participants

There were 31 participants (12 men, 19 women, 0 other gender) in this study. The average age *N* = 29 ± 9 years; 28 participants hold at least a bachelor's degree. All respondents were recruited voluntarily by an offer on social networks (Facebook, VKontakte, and Instagram). Several people reshared the original post. The post contained an email that one might use to contact the author. Some people contacted the author directly through messages on social networks. The majority of the respondents came from Facebook (≈61 per cent).

Each respondent provided at least one narrative of innocent suffering from one's own experience. Considering that each participant provided several life stories, the total number of events was *N* = 163 cases, which means that there were 5 events per respondent on average. The total time spent on the interviews was equal to 6, 924 min, which meant that the average interview lasted 223 min (3 h and 43 min). The respondents were interviewed between December 2021 and February 2022.

For the aim of the given study, this number of participants is acceptable, due to the design, methodology (grounded theory), and focus on rich data, and consistent with the number of participants in other studies (Glaser and Strauss, 1967; Henwood and Pidgeon, 2003; Hsieh and Shannon, 2005; Pals, 2006; McAdams et al., 2008; Lodi-Smith et al., 2009; Charmaz, 2014; Bengtsson, 2016). Moreover, while the number of participants is relatively low, each participant provides several examples of the experiences that occurred. Hence, the total number of cases analyzed was ≈5 times greater than the number of respondents. Furthermore, there was at least one “rich” case per respondent that contains a detailed description of an experience. These cases provided a lot of qualitative information about the phenomenon.

All respondents signed or accepted the informed consent. The study was examined and accepted by the Ethical Committee of the Saint Petersburg Psychological Society (Protocol of Acceptance N.10, Nov 25, 2021).

### 2.4. Procedure

The study consists of two parts: (1) a semi-structured interview and (2) the assessment of the reliability of the codification made. The first part is aimed to:

1. Assess the existence of the experience of innocent suffering.
2. Identify the features that distinguish this experience from others, in particular from mere “suffering”.
3. Collect demographic data and other parameters of the corresponding life event.
4. Build a prototype of innocent suffering.

The aim of the second part is to estimate the reliability of the results obtained.

#### 2.4.1. Semi-structured interview

The interview has nine blocks of questions following each other. The whole transcript of the interview is in Appendix A. Initially, a respondent is asked to provide the folk definition of innocent suffering (Block 1). It is implicitly assumed that every participant knows what this term means. Therefore, no definition or clarification of the term “innocent suffering” is provided.

Blocks 2–4 are about the experience of innocent suffering of somebody else (Block 2), somebody non-real (Block 3; characters from literature or cinema), and oneself (Block 4). Then the respondent is asked to provide a detailed description of one personal experience that happened. If there is no such event, one is allowed to choose the experience of somebody else known to the participant. If there is no such event for somebody else too, the interview is stopped.

In Block 5, the following data is collected: age of the respondent at the moment of the event, the timeline of the event, and specific features, allowing to categorize this event as critical (Holmes and Rahe, 1967; Hobson et al., 1998; Habermas and Bluck, 2000).

Block 6 is about the background of the experience, including the causes (efficient and final), and the unexpectedness, severity, and intensity of the event. Note that the participant provides this background retrospectively and subjectively from the present moment.

In Blocks 7-9, the respondent describes the coping strategies used including social support, consequences of the event, including changes in identity and behavior, and its acceptance or rejection by the participant. The last part of Block 9 is about spiritual and meaningful conclusions of the experience of innocent suffering (Baumeister, 1991; Hicks and Routledge, 2013).

As the result of the interview, the following data is gathered: (1) the list of events that represent the experiences of innocent suffering according to the respondent and (2) the detailed, “rich” description of one event with the background data.

All interviews are performed by the author of this study. The author holds a master's degree in Personality and Existential Psychology, is a counseling psychologist, and has the skills to perform the interview in a professional manner.

#### 2.4.2. Data analysis

There are two categorizations performed in the current study: (1) the identification of the features of innocent suffering and (2) the determination of the life domains related to these experiences. To find the features of the experience of innocent suffering, content analysis is used (Burnard, 1991; Graneheim and Lundman, 2004; Hsieh and Shannon, 2005; Kvale, 2007; Erlingsson and Brysiewicz, 2017). Following the methodology of grounded theory, there are three stages of coding: (1) open coding, which means the line-by-line categorization of the texts, (2) axial coding, which creates connections between categories, and (3) selective coding, which establishes links to the core variables (Glaser and Strauss, 1967; Charmaz, 2014). The procedures are repeated several times to validate and adjust the results.

To validate the categorization, three experts, who have at least a master's degree in psychology and knowledge of the related areas, were asked to confirm whether or not the given fragment of text had the given feature. Each expert signed the informed consent to participate in the study. As inputs, the condensed description of experiences is used. The question asked is the following:

Does the given fragment of text have the feature X (possible answers: 1—no, 2—possibly, yes; 3—yes, 4—definitely, yes; Streiner and Norman, 2008)

To determine whether or not the categorization is valid, the content validity ratio (CVR) is used (Lawshe, 1975; Streiner and Norman, 2008):


(1) $$\begin{array}{l} CVR=\frac{n_{e}-\frac{N}{2}}{\frac{N}{2}}, \end{array}$$

where *n*_*e*_ is the number of experts who rated 3 or more and *N* is the total number of experts. If CVR>0.99, then the identification is valid (Streiner and Norman, 2008).

To find the categories of life to which the experiences that occurred belong, the taxonomy developed by Holmes and Rahe (1967) and adjusted by Hobson et al. (1998) and Haimson et al. (2021) is used. Based on the condensed description of the experiences obtained earlier, each experience is connected to one or several categories.

Three experts, who hold at least master's degrees in psychology and gave informed consent, performed the validation and categorization of the results. If there was an agreement between all the experts and me on the categorization of an event, the categorization was considered valid. If there was an agreement between less than two experts and me, the categorization was considered invalid. The remaining cases were discussed among all experts. If there was a consensus, it was used. Otherwise, the categorization was excluded.

## 3. Results and discussion

### 3.1. Features of innocent suffering

Although no definition of innocent suffering was provided in advance, all respondents shared descriptions of the experiences that have a similar structure. It implies that the folk understanding of this phenomenon is consistent across the sample. After initial open and axial coding, six features related to the experiences of innocent suffering were identified: complexity, stability, distress, a feeling of injustice, causal incoherence, and breaks in the integrity of the narrative.

**Complexity** feature means that the experience of innocent suffering affects different levels of personality: the cognitive level (new beliefs emerge, old beliefs are rejected, and the speed and the quality of cognitive processes are changed), the affective level, mainly expressed in the form of prolonged distress, defined here as a “*negative affect and physiological reactivity*” (APA, 2021), and the existential level in the form of the actualization of the confrontation with the existential problems of death, finiteness, isolation, meaninglessness, and uncertainty (Yalom, 1980).

The distress, which every participant experienced, did not disappear due to random factors or positive events, which were presented in some texts. The background of the event and the mental state at the moment of the event (positive or negative) did not affect the intensity or duration of the experience. Therefore, one might say that the state of personality caused by the experience of innocent suffering is **stable** over time.

The experience of innocent suffering is always **negative**. There were no positive descriptions of the events that occurred. Twenty-four respondents used obscene language to describe their experiences, 7 started crying when recalling past events, and 28 respondents showed signs of discomfort describing the experience (involuntary motor movements, changes in the tone of voice, and slower pace of speech). These reactions are also valid even if events happened more than 10 or even 20 years ago.

The key differentiator of the experience of innocent suffering, which appears explicitly or implicitly, is the strong **feeling of injustice**. It might be connected to the situation as a whole (i.e., in case of violence) or some aspects of it (i.e., the intensity of stimulus in case of psychological abuse). In other words, the respondents feel that they do not deserve what has happened to them, which refers to the definition of the belief in a just world suggested by Lerner and Miller (1978).

Note that seven participants noticed that while they considered the experiences that occurred as unjust at the time of the events, now they have changed their minds. It shows that if one wants to identify this feature, one should ask about the feelings at the moment of the event not at the moment of an interview.

All respondents stated that the experience of innocent suffering was always unexpected. For a long time (from 2 months to 8 years), the participants were unable to provide a plausible explanation (for themselves) for what had happened. Hence, one might say that experiencing innocent suffering **breaks the causal coherence** of a life story because neither the event nor the experience has a plausible reason or meaning for those who experience them.

Moreover, 25 respondents claim that their life was split into “the life before the event” and “the life after the event”. From this observation and from the low level of anticipatory socialization of every respondent, it follows that the experience of innocent suffering **breaks the integrity** of a life story. Note that at the time of the study almost all respondents (26 people)^2^ integrated these experiences into their life path by creating a plausible and consistent explanation of both the event and its consequences expressed not only in the external but also the internal changes. However, the creation of a new “life model” (Grishina, 2018) required several years per participant.

To confirm the reliability of the identification of the six features described above, three experts were requested to provide their opinions. The results are in Table 1. It follows that the identification of all the features has inter-rater reliability. It should be noted that the only fluctuation is in the sixth feature, the break of the integrity of the life story, where one expert identified five events as not having this feature. In addition to random factors, one can explain this by the fact that the experts were reading the *condensed* descriptions of experiences of innocent suffering. With the condensation of meanings, some context might be lost (Kvale, 2007), which could be relevant for assessing the integrity of a life story.

**Table 1:** The results of a survey of experts on the presence of features of innocent suffering described by respondents.

***N***	**Feature**	**I**	**II**
1	Complexity	31	31
2	Stability	31	31
3	Distress	31	31
4	Feeling of injustice	30	31
5	Causal incoherence	31	31
6	Breaks of integrity	26	31

Column I has the number of events (in total—31) for which all experts rated 3 and higher. Column II indicates the number of events for which two or more experts rated 3 and higher.

To sum up, there are six features of the experience of innocent suffering appearing in every description collected: complexity, stability, distress, a feeling of injustice, causal incoherence, and breaks in the integrity of the narrative. The experts confirmed the features identified by the narrative and content analysis presented in the texts.

Based on these results, it is possible to define the experience of innocent suffering.

*Innocent suffering* is a complex and stable in-time experience expressed in distress related to the subjective feeling of injustice toward oneself or others that breaks the causal coherence and integrity of one's life story.

All six features are essential for an experience to be defined as the experience of innocent suffering.

### 3.2. Life domains

In total, there were 163 events categorized into different categories (life domains) by the experts based on the workflow described in Section 2.4.2. A total of 96 events occurred directly with the respondents. The results of the categorization are in Table 2.

**Table 2:** The number of events of innocent suffering in various categories of life.

***N***	**Category**	**Total**	**%**	**Self**	**%**
1	Health	93	39	48	35
2	Relationships	58	24	33	24
3	Family relationships	41	17	27	20
4	Identity	24	10	5	4
5	Career	7	3	11	8
6	Death	8	3	4	3
7	Financial	4	2	4	3
8	Lifestyle change	2	1	2	1
9	Legal	2	1	0	0
10	Education	1	0	2	1
11	Relocation	1	0	0	0
12	Societal	0	0	0	0

Column “Total” indicates the number of events mentioned by the respondent and categorized by experts. The same is indicated in the Column “Own” but only for events that occurred directly with the respondent. One event can be in several categories.

From Table 2, it follows that the most “popular” categories of life related to the experiences of innocent suffering described by the respondents are Health, Relationships, and Family Relationships.

For the category “Health”, the top subcategories are “abuse (including sexual abuse)”, “violent attack (including sexual assault)”, and “serious injury, accident, or physical ailment”. These subcategories describe the whole spectrum of events where the efficient cause of suffering is the status of health, both physical and mental. However, it does not mean that this is a final cause.

For example, one respondent describes the following accident: “*I remember a girl in my village who was hit by a car at a pedestrian crossing. ‘Flew out' for a year from life*.”^3^ The participant emphasizes neither the event nor its physical or psychological consequences, including the feeling of injustice (because the girl was hit at a pedestrian crossing). She emphasizes the loss of time because the girl mentioned spent a year at the hospital later.

Another very common group of events is related to cases of unexpected or unjustifiably cruel physical violence: random beating on the street, physical assault without a plausible reason, and violence in the family, especially when the victim is a child. Similarly, there are many cases of psychological violence and abuse described. Usually, it is related to child-parent relationships, school or university bullying, workplace bullying, and justified fear for one's health and wellbeing.

In the category “Relationships”, the most common subcategories of the experiences of innocent suffering are “ended serious romantic relationship”, “relationship became abusive”, and “serious argument with neighbor or friend”. Note that 17 of 19 women that participated in this study mentioned the first subcategory at least in one of the cases described, whereas for men, only 4 of 12 persons mentioned this subcategory.

The characteristic features of ending a serious romantic relationship that causes innocent suffering are unexpectedness, causelessness, and randomness (“*We have been dating for 7 years. There were [The partner had] ‘claims to the shape of my body'; quitted for no reason*”; “*A friend with whom I was close (we talked a lot for six months). Not a romantic relationship. At some point, [he] ‘disappears from [my] life'*”; ‘*It became a shock, “[like] snow on my head*^4^ *[fell]”)*, as well as serious consequences in later life (“*the hardest test ever*”; “*I had to learn again how to communicate [with other people]*”).

Another common subcategory of “Relationships” is arguing with partners or friends. The life domains of these conflicts are different: financial problems, violation of mutual agreements, or loss of romantic or friendly feelings. Often, the respondents emphasize the injustice of these events and explicitly claim that they acted in the same situations differently. The latter means that their expectations were not met.

In the category “Family Relationships”, the “popular” subcategories are “serious argument with relative” and “family betrayal”. These events are close to the corresponding events in the category “Relationships”. However, there is a group of experiences that is related to the conflicts with parents related to parental control or parenting (“*My father throw out my cat [sic]*”, “*My mother did not protect me*”).

Similar to the ending of a serious romantic relationship from the previous category is the event of divorce. There were only three participants who had such an event in their lives. The low amount of such cases is due to the sampling (the average age is low). However, all these participants emphasized that divorce was related to innocent suffering. The following feelings were reported: (self)rejection “as a wife or a woman”, betrayal of trust, injustice, loss of support, and loss of trust in the world.

Notably, one might clearly see all six features of the experiences of innocent suffering mentioned above in the event of divorce and ending a serious romantic relationship:

1. Such experiences are complex and affect all levels (physical, cognitive, affective, and existential).
2. They last for years. The consequences of these events may last even more.
3. These events cause distress, in two out of three cases, requiring medical treatment and long-term psychotherapy.
4. The experiences include the strong feeling of injustice expressed in the betrayal of trust.
5. Such events are not causally linked even in situations where it was clear in advance (with a retrospective assessment) that separation or divorce was imminent.
6. These experiences break the integrity of the life story. All the respondents split their life into “the life before” and “the life after” the corresponding event.

### 3.3. Ending innocent suffering

The turning point of the experience of innocent suffering that starts the restoration of causal coherence and integrity of life story is the time when respondents become active and accept the responsibility for their lives. The coping strategies differ: switching focus to work, job change, starting working with a psychologist, self-development, self-socialization, or environmental changes. This leads to the construction of the meaning of the situation that happened.

The majority of respondents (*N* = 19) stated that the experience of innocent suffering strengthened them. One of the respondents quoted Nietzsche: “*What does not kill us, makes us stronger*” (Nietzsche and Hollingdale, 2003). Implicitly, the declaration of this meaning implies awareness and acceptance of personality changes related to the event. It restores the causal coherence of the respondent's life story.

Another common meaning (*N* = 9) is maturity. The participants acknowledged that they got life experience that allowed them to become more mature and wiser and not act in some situations as a child. The rest of the respondents (*N* = 3) had an ongoing experience. Hence, it is impossible to identify its meaning because the event is not completed.

To sum up, every participant mentioned explicitly one of the two meanings above as the result of the experience of innocent suffering. It appears that the end of the meaning-making process restores, finally, the causal coherence and integrity of the life story and completes the event. Interestingly, the heroes of the poems mentioned in the Introduction found the same meaning (Smirnov, 2021). Surprisingly, nobody rejects (like Job) the belief in a just world. This confirms the hypothesis suggested by Lerner and Miller (1978) that this belief is a real psychological need not just a cognitive distortion (Hafer and Sutton, 2016).

### 3.4. A prototype

Based on the results, it is possible to create a prototype of the experience of innocent suffering. Usually, it starts with a trigger, which is a formal *casus belli*. The trigger can be a random event (i.e., random reading of messages of a partner and random assault on the street), something that has been brewing for a long time and has finally come (for example, when the relationships between two people were getting worse and the partner did not congratulate the respondent on respondent's birthday or another significant date), or a state of extreme physical or mental exhaustion (long lack of sleep; long illness).

Such an event always happens unexpectedly. Note that unexpectedness is actual at the time of the event. Later, one might change one's mind. The very first reaction to the unexpected event is dissonance. In the case of innocent suffering, the intensity is high because of the feeling of injustice that leads to the causal incoherence of the participant's life story. This experience may lead to a confrontation with one of the existential problems: death, isolation, meaninglessness, or freedom (Yalom, 1980). The restoration is possible only if one creates (or finds) meaning in the situation. The most common meanings are strengthening one's personality, maturing, and getting new life experiences.

When one reveals and accepts the meaning, the integrity of the life story is restored. The frustration caused by injustice decreases because of the secondary gain and new opportunities. Sometimes, it appears spontaneously. Sometimes, a respondent has to invest conscious time and resources into this process.

Overall, the experience of innocent suffering embeds into one's life path that restores the causal coherence of the life story. This might happen because, for several reasons, one might (1) reject the belief in a just world, (2) find meaning in suffering, and (3) find the real cause of the event.

**Figure 1:**
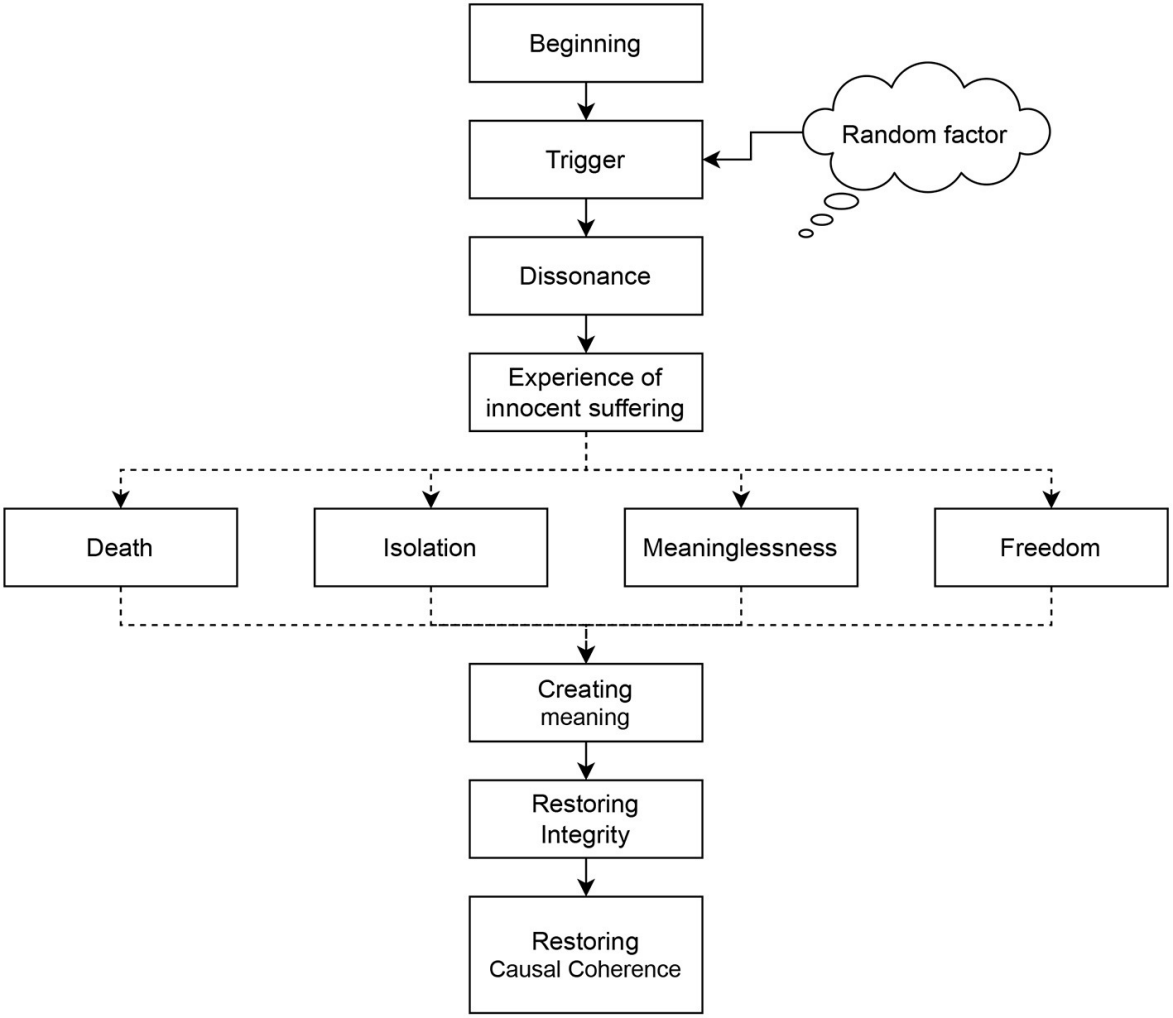
The prototype of an experience of innocent suffering. Four items in the middle of the figure (Death, Isolation, Meaninglessness, and Freedom) represent four existential problems as they are defined by Yalom (1980).

All steps described above are in Figure 1.

## 4. Limitations

It should be noted that the results obtained have some biases, which prevent generalization. For example, the categories of life related to innocent suffering may differ in other samples. The known biases are the following:

1. Almost all the respondents (30 of 31) have a bachelor's degree or a higher level of education.
2. The total number of respondents (31) is relatively small for any statistically significant inferences.
3. The age of the participants does not exceed 41 years.
4. All the respondents live in large cities.
5. Social statuses of participants are close to each other.
6. All the respondents live in the same country.
7. The workflow of recruiting is not fully random.
8. All interviews are performed by the author.

While one might argue that the results obtained seem to be the same for other samples, they should be further verified empirically.

## Data availability statement

The datasets presented in this article are not readily available because since the participants shared very personal experiences of their lives, the special confidentiality policy is applied. Requests to access the datasets should be directed to ES, smirik@gmail.com.

## Ethics statement

The studies involving human participants were reviewed and approved by Ethical Committee of St. Petersburg Psychological Society. The patients/participants provided their written informed consent to participate in this study.

## Author contributions

The author confirms being the sole contributor of this work and has approved it for publication.

## Footnotes

^1^The heroes of the ancient poems mentioned are always men. Therefore, the word “he” is used.

^2^4 respondents described the experiences that were actual and ongoing at the time of the study. Therefore, it is incorrect to estimate whether or not they have integrated these experiences into their lives.

^3^Hereinafter, all quotes are translated by me.

^4^An idiom, which means something extremely unexpected.

## Appendix

### Appendix A. The questions of the interview

1. Could you please tell me what “innocent suffering” means to you?
2. Did you remember a situation when you have seen a person who experienced innocent suffering? If the answer is “yes”, could you please describe this situation?
3. Could you please name any character (from a film, a book, etc.) that experienced innocent suffering?
4. Did you experience any suffering, which you did not deserve (based on your feelings at that moment)? If the answer is “yes”, could you please describe this situation? If the answer is “no”, could you please remember a situation, when you suffered a lot (does not matter whether these sufferings were innocent)?
5. Questions about the situation:
  a. General: your age and overall mental state at that time.
  b. The features that identify the critical life event (CLE):
  (1) When the event happened, did you know what to do?
  (2) What feelings and emotions did you have? Could you please compare them with the regular (at that time)?
  (3) How important is this event in your life (from 0 to 200)?

6. Background:
  a. What do you think was the reason for the sufferings?
  b. What was the trigger that “launched” the sequence of events?
  c. How unexpected was the event?
  d. What was the most difficult for you?
  e. When did the situation start getting better?
  f. Could you please describe how the intensity of the suffering has changed over time?
  g. Did you have any health or mental diseases at the time of the event (including allergies, etc)?

7. Dreams and Signs:
  a. Did you remember any dreams from that period of time? If the answer is “yes”, could you please describe these dreams?
  b. Did you notice any “signs of the universe”? Maybe you often listened to a certain song or read a specific book.

8. Coping:
  a. How did you manage this situation?
  b. How did your family and friends react to the situation?

9. Effects of CLE:
  a. If you could go back to the moment of the event, would you have done anything differently? If so, what?
  b. If you could go back in time for a while before the event, would you change anything? How would this affect you in the present?
  c. What has changed in you as a result of these sufferings?
  d. Have these changes been expressed in any way in the external world (environment, status, circle of communication, etc.)?
  e. What is the meaning of these sufferings for you?
  f. If there was a God (or exists, if you believe in Him) in the world, what do you think is the purpose of such suffering?
